# Retinal capillary rarefaction in patients with untreated mild-moderate hypertension

**DOI:** 10.1186/s12872-017-0732-x

**Published:** 2017-12-21

**Authors:** Agnes J. Bosch, Joanna M. Harazny, Iris Kistner, Stefanie Friedrich, Joanna Wojtkiewicz, Roland E. Schmieder

**Affiliations:** 1 Department of Nephrology and Hypertension, Friedrich-Alexander-University Erlangen-Nürnberg (FAU), Ulmenweg 18, 91054 Erlangen, Germany; 2 Department of Pathophysiology, University of Warmia and Mazury, Olsztyn, Poland

**Keywords:** Capillary rarefaction, Hypertension, Retina, Microcirculation, Capillary density

## Abstract

**Background:**

Microvascular rarefaction influences peripheral vascular resistance, perfusion and metabolism by affecting blood pressure and flow pattern. In hypertension microvascular rarefaction has been described in experimental animal studies as well as in capillaroscopy of skin and biopsies of muscle tissue in patients. Retinal circulation mirrors cerebral microcirculation and allows non-invasive investigations. We compared capillary rarefaction of retinal vessels in hypertensive versus normotensive subjects.

**Methods:**

In this study retinal capillary rarefaction in 70 patients with long time (more than 67 month of disease duration) and 64 patients with short time hypertension stage 1 or 2 has been compared to 55 healthy control subjects, who participated in clinical trials in our Clinical Research Center (www.clinicaltrials.gov: NCT01318395, NCT00627952, NCT00152698, NCT01319344). Retinal vascular parameters have been measured non-invasively and in vivo in perfusion image by scanning laser Doppler flowmetry (Heidelberg Engineering, Germany). Capillary rarefaction was assessed by capillary area (CapA) (in pixel-number) and intercapillary distance (ICD) (in μm). Additionally retinal capillary flow (RCF) was measured.

**Results:**

ICD was greater in the long time hypertensive group compared to healthy individuals (24.2 ± 6.3 μm vs 20.1 ± 4.2 μm, *p* = 0.001) and compared to short time hypertensive patients (22.2 ± 5.2 μm, *p* = 0.020). Long time hypertensive patients showed less CapA compared to healthy people (1462 ± 690 vs 1821 ± 652, *p* = 0.005). Accordingly, RCF was significantly lower in the long time hypertensive group compared to the healthy control group (282 ± 70 AU vs 314 ± 60 AU, *p* = 0.032). Our data indicate a lower level of retinal capillary density in hypertensive patients, especially in those with long time hypertension.

**Conclusion:**

Patients with hypertension stage 1 or 2 showed retinal capillary rarefaction in comparison to healthy normotensive subjects. Retinal capillary rarefaction was intensified with duration of disease.

**Electronic supplementary material:**

The online version of this article (10.1186/s12872-017-0732-x) contains supplementary material, which is available to authorized users.

## Background

In patients with primary hypertension the microcirculation is characterized by substantial structural and functional changes [1]. These changes identified as signs of end-organ damage and caused by hypertension are known to predict cardiovascular events [2]. A decreased number of arterioles and capillaries is called microvascular rarefaction and has been already described in various experimental animal studies [3, 4] as well as in capillaroscopy of skin [5–7] and biopsies of muscle tissue [8] in patients with primary hypertension. An inverse correlation between skin capillary density and blood pressure (BP) has been identified in hypertensive patients [9, 10]. Decreased capillary density caused e.g. by hypertension influences the flow pattern in the microvascular bed. This results in a non-uniform blood flow distribution among exchange vessels [6]. Thus, capillary and arterial rarefaction, by affecting both pressure and flow pattern, may have consequences for perfusion and metabolism besides the known influence on peripheral vascular resistance [6, 11].

Structural capillary rarefaction originates from anatomical absence of capillaries [1]. Retinal vessels are located in the inner layers of the retina and pericytes surround endothelial cells at the perivascular interface. Pericytes stabilize blood vessels and maintain constant communication with endothelial cells [12]. A loss of pericytes results in capillary instability, pathological angiogenesis and rarefaction [13]. On the other hand, enhanced contractile activity of pericytes causes capillary contraction (no-reflow phenomenon) and entrapment of erythrocytes [12]. This phenomenon, called functional non-perfusion, is defined as reduction of the number of spontaneously perfused capillaries and also contributes to capillary rarefaction in addition to structural components [10]. In skin capillaries functional rarefaction measurements have been shown to correlate inversely with endothelial function [14]. Endothelial injury causes decreased bioavailability of nitric oxide and reduced arteriolar vasodilatory capacity. As a result blood flow to the capillaries is ultimately reduced [15]. Functional rarefaction was found in cerebral capillaries in hypertensive animal models [16, 17]. Hypertension is known to be associated with an over activity of the renin angiotensin system. This over activity has been shown to cause vasoconstriction also in the cerebral blood supply, which was associated with an increased susceptibility to stroke and dementia [16]. Measurement of cerebral arterioles and capillaries in humans is difficult and invasive. There is rising evidence that retinal vessels may mirror the cerebral and systemic microcirculation [18].

Changes in small artery structure and function can also be assessed in vivo, reliably and non-invasively in retinal vessels using scanning laser Doppler flowmetry (SLDF) [19]. Retinal arteriolar wall-to-lumen ratio (WLR) has been shown to be a feature of microvascular target organ damage in hypertension. Whereas hypertension has been found to be associated with increased retinal WLR [20], there is a lack of data investigating retinal capillary density in hypertensive patients. SLDF of retinal vessels allows the investigation of capillary area (CapA), intercapillary distance (ICD) and retinal capillary flow (RCF) based on analysis of perfusion images. One of our previously analyses showed retinal capillary rarefaction in patients with diabetes mellitus type two compared to healthy individuals [21]. Patients with hypertension have been shown to present similar values of CapA und ICD when compared to patients with diabetes mellitus type two [21]. Increasing duration of hypertension has been previously shown to be associated with a deterioration of postocclusive hyperemia [22]. As retinal arteriolar changes, such as WLR, have been shown to be associated with disease duration in different study populations [23], we now investigated capillary rarefaction in patients with short time and long time hypertension stage 1 or 2 and compared our results to normotensive control subjects.

## Methods

### Study design

This is a retrospective comparative study comprising data of patients and controls who participated in previous randomized, double blind, parallel, mono-center clinical trials in our Clinical Research Unit of the Department of Nephrology and Hypertension, University of Erlangen-Nürnberg, Germany (www.clinicaltrials.gov: NCT01318395, NCT00627952, NCT00152698, NCT01319344). We consecutively enrolled eligible subjects in the area of Erlangen-Nürnberg, Germany, who had been recruited by advertisements in newspapers. Before study inclusion written informed consent was obtained in each patient. The studies were conducted in accordance with the Declaration of Helsinki and the principles of good clinical practice guidelines. The study protocol of each trial was approved by the Local Ethics Committee (University of Erlangen-Nürnberg).

### Study population

Female and male, non-smoking, Caucasian patients with primary hypertension stage 1 or 2 (systolic BP 140–179 mmHg and diastolic BP 90–109 mmHg) with a trough mean sitting systolic BP ≥ 140 mmHg and/or diastolic BP ≥ 90 mmHg and healthy male and female, non-smoking, Caucasian subjects were included. Hypertensive patients were categorized according to median disease duration (67 month) and separated in two groups (short time and long time hypertensives). Patients had to present with excellent SLDF images fulfilling the pixel criteria for inclusion. The “pixel criteria” requires an excellent SLDF image [24] with less than three saccades to clearly distinguish every pixel from the next one, which is necessary to identify ICD and CapA. All healthy individuals and hundred thirty-four out of 158 hypertensive patients fulfilled pixel criteria for inclusion. Thus, baseline data of 55 healthy individuals and 70 short time as well as 64 long time hypertensive patients were included. The healthy reference study population has been previously described by our study group [21].

Office blood pressure (BP) was taken unattended with an automated device at screening visit and additionally at the same time as SLDF measurement was performed. BP measurements have been performed according to guidelines recommendations and in a standardized fashion. Office BP was measured three times in a quite sitting position. The mean value of these three measurements was calculated and defined as the trough mean sitting BP. 24 h ambulatory BP was measured with the Mobil-O-Graph I.E.M. System, for which good validity and reliability has been demonstrated [25, 26].

Homogeneity of the hypertensive study population was ensured by in- and excluding patients using the same criteria. (www.clinicaltrials.gov NCT01318395, NCT00627952) Main inclusion criteria for both clinical trials were male or female persons aged 18 years or older with mild to moderate uncomplicated essential hypertension with a trough mean sitting DBP ≥ 90 mmHg and/or SBP ≥ 140 mmHg or pretreated arterial hypertension. Main exclusion criteria for both clinical trials were secondary hypertension and severe essential hypertension (systolic BP ≥ 180 mmHg and/or diastolic BP ≥ 110 mmHg). Patients of the hypertensive group were previously treated for hypertension and received a wash-out period of 4 weeks antihypertensive medication prior to SLDF measurement.

Homogeneity of the healthy non-smoking control group was ensured by including healthy Caucasian individuals older than 18 years without any cardiovascular disease and without any other significant disease. Healthy individuals from the control group of the following clinical trial were included: www.clinicaltrials.gov NCT00152698. We also included healthy individuals who have been screened for the following clinical trial www.clinicaltrials.gov NCT01319344, but did not meet obesity, triglyceride, HDL and fasting blood glucose criteria for metabolic syndrome.

### Retinal arteriolar structure and retinal capillary flow

SLDF at 670 nm (Heidelberg Retina Flowmeter, Heidelberg Engineering, Germany) was used to determine the vascular wall lumen diameters [19, 27]. For perfusion analysis, capillary vessels with diameter ≤ 20 μm were selected and blood flow calculation was performed using the automatic full field perfusion image analyser (AFFPIA) [24]. The method of SLDF and AFFPIA have been extensively described in previous studies [28]. In brief, outer arteriolar diameter (OD) was measured in reflection images, and inner (lumen) diameter (ID) was assessed in perfusion images (software version SLDF 4.0). WLR was determined using the formula (OD-ID)/ID, wall thickness (WT) was calculated using the formula OD – ID/2 and cross-sectional area (CA) was determined using the formula 3.14 x (OD [2] – ID [2])/4 [27, 29]. Measurements of perfusion, distance and area were used to determine capillary density. Reliability of SLDF measurement has been previously shown to be fair (coefficient of variation <10%) [27, 30].

### Retinal intercapillary distance and area

Measurement of ICD and CapA is based on perfusion image of pixels and has been previously described in detail [28]. In brief, ICD was defined as distance between any pixel (smallest dot of optic solution, where flow can be detected) outside and the next pixel inside the vessel and is given in μm. CapA was defined as area with predominance of vessels with ≤20 μm diameter and is given in number of pixels. Reliability of measurements have been previously shown to be fair (coefficient of variation <10%) [28].

### Statistical methods

Normal distribution of data was analyzed using Kolmogorov-Smirnov test. In addition, Levene-test has been performed to check homogeneity of variance. For each variable showing Levene *p* values ≤ 0.05 data were considered as unbalanced and therefore not normal distributed. Normally distributed data were compared by unpaired student t-tests and ANOVA, respectively. Data are given as mean ± SD in tables. For not normally distributed parameters Mann-Whitney-U-Test was used for further analysis. Two-tailed values of *p* < 0.05 were considered statistically significant. Multiple regression analysis was used to identify confounding variables and subsequently adjustment was made for variables with significant differences between both groups such as: Gender, BMI, systolic office BP, Serum-creatinine, HDL-cholesterol (model 1) as well as additionally for age, LDL-cholesterol and ex-smoking habit (model 2). Multivariate regression analysis was also made using model 3, which includes adjustment for systolic 24 h ambulatory blood pressure instead of office blood pressure and the other factors included in model 1. Bivariant correlation analyses were performed using Pearson’s test. Partial correlation analyses were performed with adjustment for model 1, 2 and 3. Reliability was evaluated using coefficient of variation (CV = SD*100/mean) and Cronbach’s alpha. All analyses were performed using IBM SPSS Statistics 22 (SPSS Inc., Chicago, IL).

## Results

### Study population

70 male and female patients aged 52 ± 9.3 years with short time hypertension (less than 67 month disease duration (median of total hypertensive group)) and 64 long time male and female hypertensive patients (more than 67 month disease duration) aged 51 ± 13 years were included. All hypertensive patients were non-smoking, had diagnosed uncomplicated primary hypertension stage 1 or 2, without evidence of severe end-organ-damage. The hypertensive study population was compared to 55 non-smoking, healthy normotensive control subjects aged 53 ± 13 years. All patients of the hypertensive group received 4 weeks washout of their respective antihypertensive medication prior to SLDF measurements [23]. There were more female persons in the healthy control group (*p* < 0.001) and body mass index (BMI) was lower in the healthy group (*p* = 0.011) compared to the hypertensive population. There were also significant differences in HDL-, and total cholesterol, as well as serum-creatinine (Table 1). OD (*p* = 0.038) and ID (*p* = 0.004) were significantly smaller in hypertensive patients and long time hypertensive patients (OD *p* = 0.052, ID *p* = 0.010) compared to the healthy control group. There was no difference in WT, WLR and CA between the total hypertensive and healthy group as well as between the long time hypertensive and healthy group (Table 1).

**Table 1:** Study population

	Short time hypertensive patients (*n* = 70)	Long time hypertensive patients (*n* = 64)	Healthy control subjects (*n* = 55)		ANOVA results *p*-value
Clinical characteristics					
Age (years)	52 ± 9.3	51 ± 13	53 ± 13		*p* = 0.585
BMI (kg/m^2^)	28.3 (25–31)	27.8 (26–30)	25.3 (23–29)		*p = 0.011*
Sex (male/female)	46/24	53/11	19/36		*p < 0.001*
Systolic Office BP (mmHg)	146 ± 12	147 ± 11	124 ± 12		*p < 0.001*
Diastolic Office BP (mmHg)	90 ± 8.6	89 ± 9.1	79 ± 9.1		*p < 0.001*
HR (bpm)	73 ± 8.6	73 ± 7.9	70 ± 13		*p* = 0.739
Systolic 24 h Ambulatory BP (mmHg)	142 ± 10.2	148 ± 9.7	120 ± 9.6		*p < 0.001*
Diastolic 24 h Ambulatory BP (mmHg)	89 ± 9.4	92 ± 9.9	75 ± 4.1		*p < 0.001*
Serum creatinine (mmol/L)	0.077 ± 0.02	0.080 ± 0.01	0.073 ± 0.02		*p = 0.013*
Cholesterol (mmol/L)	5.75 ± 0.98	5.57 ± 0.98	6.16 ± 1.19		*p < 0.001*
LDL (mmol/L)	3.89 ± 0.96	3.76 ± 0.98	4.04 ± 0.93		*p* = 0.138
HDL (mmol/L)	1.39 ± 0.33	1.29 ± 0.27	1.63 ± 0.34		*p < 0.001*
Plasma glucose (mmol/L)	5.05 ± 0.54	5.11 ± 0.66	4.83 ± 0.93		*p* = 0.306
Retinal parameters					
	Short time Hypertensive patient	Long time Hypertensive patients	Healthy control subjects	*p*-value healthy vs hypertensive	*p*-value healthy vs long time hypertensive
ICD (μm)	22.2 ± 5.2	24.2 ± 6.3	20.1 ± 4.2	*p = 0.001*	*p < 0.001*
CapA (−)	1643 ± 602	1462 ± 690	1821 ± 651	*p = 0.013*	*p = 0.005*
RCF (AU)	299 ± 72	282 ± 70	314 ± 60	*p = 0.032*	*p = 0.010*
OD (μm)	102 ± 12	102 ± 15	107 ± 11	*p = 0.038*	*p = 0.052*
ID (μm)	76.1 ± 7.1	75.5 ± 8.5	79.6 ± 8.0	*p = 0.004*	*p = 0.010*
WT (μm)	13.2 ± 3.7	13.2 ± 4.4	13.6 ± 3.8	*p* = 0.552	*p* = 0.614
WLR (−)	0.345 ± 0.08	0.346 ± 0.10	0.352 ± 0.11	*p* = 0.693	*p* = 0.769
CA (μm [2])	3773 ± 1408	3806 ± 1698	3963 ± 1489	*p* = 0.540	*p* = 0.646

Data are given as mean ± SD, duration of disease, BMI is given as median and interquartile range, *BMI* body mass index, *BP* blood pressure, *HR* heart rate, *LDL* low density lipids, *HDL* high density lipids, *RCF* retinal capillary flow, *ICD* intercapillary distance, *CapA* capillary area, *OD* outer diameter retinal arteriole, *ID* inner diameter retinal arteriole, *WT* wall thickness retinal arteriole, *WLR* wall to lumen ratio, *CA* cross sectional area

The data for the healthy control subjects have been previously described by our study group [21]

### Retinal capillary density and retinal capillary flow

To eliminate potentially confounding differences in CV-risk factors between the groups compared, adjustment was made for gender, BMI, systolic office BP, serum-creatinine and HDL-cholesterol (model 1) and in addition for age, LDL-cholesterol and ex-smoking habit. Since 24 h ambulatory BP is considered the gold standard of BP measurement, a third multivariate regression model has been established adjusting for systolic 24 h ambulatory BP instead of office blood pressure. The results of all multivariate regression models are shown in Table 2.

**Table 2:** Retinal parameters after multivariate adjustment for model 1–3

Retinal parameters						
	Short time Hypertensive	Long time Hypertensive	Healthy control subjects	*p*-value model 1	*p*-value model 2	*p*-value model 3
ICD (μm)	22.2 ± 5.2	24.2 ± 6.3	20.1 ± 4.2	*p* = 0.011	*p* = 0.008	*p* = 0.035
CapA (−)	1643 ± 602	1462 ± 690	1821 ± 651	*p* = 0.001	*p* = 0.001	*p* = 0.010
RCF (AU)	299 ± 72	282 ± 70	314 ± 60	*p* = 0.010	*p* = 0.009	*p* = 0.071

Data are given as mean ± SD, *ICD* intercapillary distance, *CapA* capillary area, *RCF* retinal capillary flow, adjusted *p*-value: total hypertensive group versus healthy subjects

The data for the healthy control subjects have been previously described by our study group [21]

Model 1 includes: gender, BMI, systolic office BP, serum-creatinine and HDL-cholesterol

Model 2 includes: gender, BMI, systolic office BP, serum-creatinine and HDL-cholesterol, age, LDL- cholesterol and ex-smoking habit

Model 3 includes: gender, BMI, serum creatinine, HDL-cholesterol, systolic 24 h ambulatory BP

Measurements of ICD and CapA were used to determine capillary density. When comparing the total hypertensive to the healthy control group ICD was greater (*p* = 0.001) and CapA smaller (*p* = 0.013) in the hypertensive group (Table 1). Both parameters remained significant between the two groups after multivariate adjustment (Table 2). Thus, our data indicate a lower level of capillary density in hypertensive patients. RCF was significantly smaller in the total hypertensive group compared to the healthy control (*p* = 0.032) and there was a trend toward significance after adjustment for model 3 (see Table 2).

When comparing the long time hypertensive group to healthy individuals ICD remained greater (*p* < 0.001) and CapA smaller (*p* = 0.005) indicating capillary rarefaction. Also RCF was significantly smaller in the long time hypertensive group compared to healthy individuals (*p* = 0.010) (Table 1). Patients with short time hypertension showed still greater ICD (*p* = 0.020), but no difference in CapA (*p* = 0.122) and RCF (*p* = 0.241) when compared to healthy individuals. When compared to patients with long time hypertension short time hypertensive individuals showed smaller ICD values (*p* = 0.047), but no difference in CapA (*p* = 0.112) and RCF (*p* = 0.161).

Separation of the hypertensive study population according to median of 24 h ambulatory systolic or diastolic blood pressure did not reveal a clear differing signal in WLR and capillary rarefaction between the groups (data not shown).

### Retinal capillary density and wall-to-lumen ratio

In the total study population there was a correlation between CapA and WLR

(*r* = −0.173, *p* = 0.021) and a correlation between ICD and WLR (*r* = 0,138, *p* = 0.063), which persisted after multivariate adjustment (Table 3).

**Table 3:** Partial correlation after adjustment for model 1–3

Adjustment for model 1			
	Total study population	Hypertensive patients	Healthy control subjects
ICD (μm) and WLR (−)	*r* = 0.146	*r* = 0.192	*r* = −0.150
	*p* = 0.060	*p* = 0.035	*p* = 0.343
CapA (−) and WLR (−)	*r* = −0.195	*r* = −0.189	*r* = −0.128
	*p* = 0.011	*p* = 0.038	*p* = 0.419
ICD (μm) and HDL (mmol/L)	*r* = −0.187	*r* = −0.112	*r* = 0.010
	*p* = 0.014	*p* = 0.278	*p* = 0.964
CapA (−) and HDL (mmol/L)	*r* = 0.156	*r* = 0.069	*r* = 0.027
	*p* = 0.040	*p* = 0.502	*p* = 0.902
Adjustment for model 2			
	Total study population	Hypertensive patients	Healthy control subjects
ICD (μm) and WLR (−)	*r* = 0.192	*r* = 0.254	*r* = 0.027
	p = 0.014	p = 0.006	*p* = 0.873
CapA (−) and WLR (−)	*r* = −0.229	*r* = −0.276	*r* = −0.175
	p = 0.003	*p* = 0.003	*p* = 0.293
ICD (μm) and HDL (mmol/L)	*r* = −0.172	*r* = −0.083	*r* = −0.032
	*p* = 0.025	*p* = 0.428	*p* = 0.889
CapA (−) and HDL (mmol/L)	*r* = 0.148	*r* = 0.026	*r* = 0.098
	*p* = 0.050	*p* = 0.803	*p* = 0.672
ICD (μm) and systolic 24ABP	*r* = 0.253		
	*p* = 0.009		
ICD (μm) and diastolic 24ABP	*r* = 0.190		
	*p* = 0.050		
ICD (μm) and systolic OBP	*r* = 0.143		
	*p* = 0.060		
Adjustment for model 3			
ICD (μm) and WLR (−)	*r* = 0.227	*r* = 0.231	*r* = 0.133
	*p* = 0.018	*p* = 0.032	*p* = 0.611
CapA (−) and WLR (−)	*r* = −0.256	*r* = −0.212	*r* = −0.431
	*p* = 0.007	*p* = 0.048	*p* = 0.084
ICD (μm) and HDL (mmol/L)	*r* = −0.243	*r* = −0.210	*r* = −0.252
	*p* = 0.011	*p* = 0.051	*p* = 0.329
CapA (−) and HDL (mmol/L)	*r* = 0.224	*r* = 0.148	*r* = 0.387
	*p* = 0.020	*p* = 0.172	*p* = 0.125

*ICD* intercapillary distance, *CapA* capillary area, *WLR* wall to lumen ratio, *HDL* high density lipids, *ABP* ambulatory blood pressure, *OBP* office blood pressure.

The data for the healthy control subjects have been previously described by our study group [21]

Model 1 includes: gender, BMI, systolic office BP, serum-creatinine and HDL-cholesterol

Model 2 includes: gender, BMI, systolic office BP, serum-creatinine and HDL-cholesterol, age, LDL- cholesterol and ex-smoking habit

Model 3 includes: gender, BMI, serum creatinine, HDL-cholesterol, systolic 24 h ambulatory BP

**Fig. 1:**
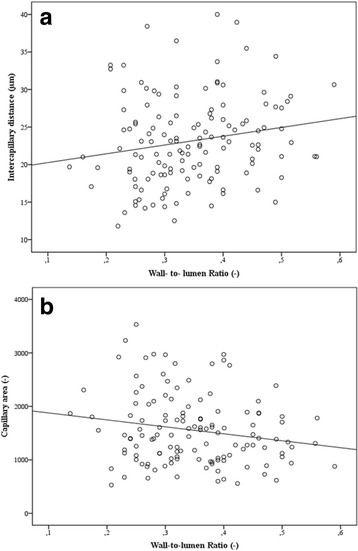
Correlation of retinal capillary density and wall-to-lumen ratio in hypertensive patients. **1a**: Intercapillary distance and wall-to-lumen ratio (*r* = 0.185, *p* = 0.033). **1b**: Capillary area and wall-to-lumen ratio (*r* = −0.185, *p* = 0.035)

Capillary rarefaction correlated with WLR in the hypertensive study population (ICD: *r* = 0.185, *p* = 0.033, CapA: *r* = −0.185, *p* = 0.035) (Fig. 1). Capillary rarefaction also significantly correlated with WLR in the hypertensive study population after adjustment for model 1–3 (Table 3).

There was no correlation between capillary rarefaction and WLR in the healthy population (ICD: *r* = 0.043, *p* = 0.770, CapA: *r* = −0.158, *p* = 0.287).

### Retinal capillary density and blood pressure

**Fig. 2:**
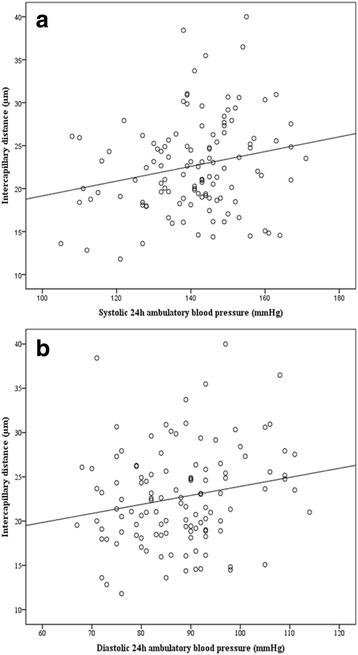
Correlation of retinal Intercapillary distance and 24 h ambulatory BP. **2a**: Intercapillary distance and systolic 24 h ambulatory BP (*r* = 0.238, *p* = 0.010). **2b**: Intercapillary distance and diastolic 24 h ambulatory BP (*r* = 0.219, *p* = 0.018)

In the total study population, there was a correlation between ICD and systolic 24 h BP (*r* = 0.238, *p* = 0.010) and diastolic 24 h BP (ICD: *r* = 0.219, *p* = 0.018) (Fig. 2) (for adjustment except BP see Table 3). There was a trend towards a correlation between ICD and systolic office BP (*r* = 0.134, *p* = 0.067) (for adjustment except BP see Table 3). No correlation was found between ICD and diastolic office BP. Furthermore no correlation was present between CapA and systolic as well as diastolic office BP. Separate analysis of the hypertensive and the healthy study population revealed no correlation of retinal capillary density with office or ambulatory BP, respectively.

**Fig. 3:**
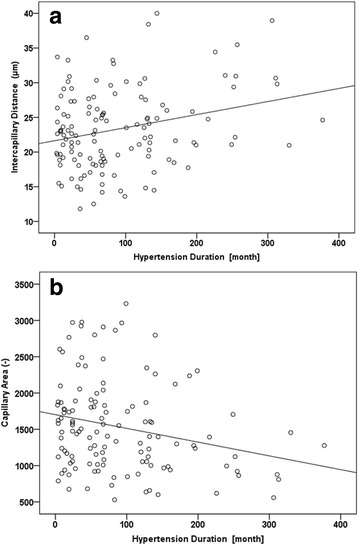
Correlation of capillary density and duration of hypertension. **3a**: Intercapillary distance and duration of hypertension (*r* = 0.263, *p* = 0.002). **3a**: Capillary area and duration of hypertension (*r* = −0.243, *p* = 0.006)

In the hypertensive group, there was a correlation between ICD and hypertension duration (*r* = 0.263, *p* = 0.002), which persisted after full adjustment (model 2) (*r* = 0.213, *p* = 0.045) and an inverse correlation between Cap A and hypertension duration (*r* = −0.243, *p* = 0.006), which remained as a trend after full adjustment (*r* = −0.194, *p* = 0.068) (Fig. 3). Hence, our data indicate a possible association between duration of hypertension and retinal capillary rarefaction.

### Capillary density and lipid metabolism

**Fig. 4:**
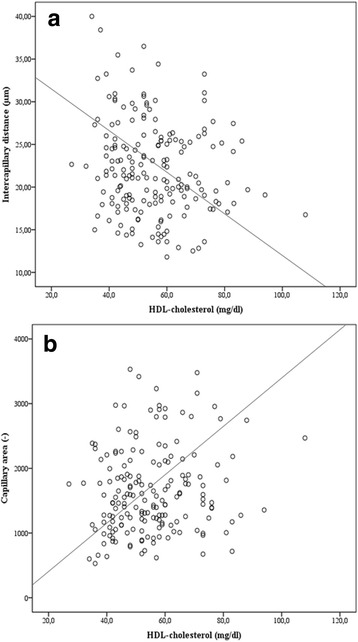
Correlation of capillary density and HDL. **4a**: Intercapillary distance and HDL-cholesterol (*r* = −0.174, p = 0.018). **4b**: Capillary area and HDL-cholesterol (*r* = 0.136, *p* = 0.070)

ICD correlated with HDL-cholesterol level inversely in the total study population.

(*r* = −0.174, p = 0.018) (Fig. 4). There was also a trend towards a correlation between CapA (*r* = 0.136, *p* = 0.070) and HDL-cholesterol. After adjustment for model 1–3 except HDL, the correlation of CapA with HDL-cholesterol remained significant (see Table 3). Hence, our data indicate an association between capillary rarefaction and serum HDL-cholesterol. There were no significant correlations of parameters of capillary density with LDL-cholesterol levels.

## Discussion

As to our knowledge this is the first in-vivo description of retinal capillary rarefaction in patients with short time and long time hypertension - a non-invasive approach to get an inside of the systemic and cerebral microcirculation. Our measurements of retinal capillary rarefaction (ICD and CapA) and RCF indicate a lower level of retinal capillary density in hypertensive patients compared to normotensive individuals, which is intensified in patients with long time hypertension. This is in accordance with the result of previous studies, which analyzed capillary density in the skin [5–7] and muscle tissue [8] of hypertensive patients.

In routine, studies under standardized biological conditions SLDF measurements previously showed high reliability (CV <10% for all parameters, except of CA with 12.5%). [27, 31, 32], We also demonstrated good reliability of ICD and CapA in one of our preexperiments (CV ≤ 10% for ICD and CapA) [28].

Structural vessel measurements of retinal arterioles have been previously shown to provide information similar to that obtained in subcutaneous small arteries [18], even though retinal vessels belong to the cerebro-vascular perfusion area and therefore may mirror cerebral vessel pathophysiology in addition to microcirculation in general [33]. WLR of retinal arteries and media-to-lumen ratio of subcutaneous small arteries in normotensive and hypertensive patients have been previously shown to correlate [18]. Visualization of skin capillaries was performed with either intravenous fluorescent dyes or intravital video-microscopy. The former has the disadvantage of being invasive [10]. Retinal vessels have the advantage to be easy accessible to direct noninvasive visualization. Measurement of capillary density depends on perfusion of capillaries and therefore mirrors structural and functional retinal rarefaction. It can be considered as an advantage of SLDF compared to other optic systems such as Adaptive optics imaging, that SLDF also allows perfusion dependent assessment of vessels and therefore analysis of capillaries. Retinal vessel changes, especially changes in retinal arteriolar WLR, have been shown to be related to other signs of end-organ damage such as urinary albumin-to-creatinine ratio [34].

Measurement of retinal arteries and capillaries using SLDF takes approximately fifteen minutes in each patient and does not require the use of mydriatic agents. We therefore consider the method as potentially applicable in routine clinical practice in patients with hypertension at risk to develop end-organ damage. Unfortunately the needed devices are not yet routinely available.

It is well known that the spatial pattern of flow in the microvascular bed is affected by decreased capillary density [6]. This has been further stressed in our study showing a lower RCF in hypertensive patients with capillary rarefaction in comparison with normotensive patients. In hypertensive patients RCF has been shown to be negatively related to WLR, a distinctive parameter of vascular end-organ damage, independently of cardiovascular risk factors [35]. There are two clinical studies indicating no difference in basal RCF in normotensive and hypertensive patients stage 1 or 2, which can possibly be explained by huge differences in age (patients in our clinical trial were 16 years older), smoking habit (we included patients who never smoked) and choice of different spots for SLDF measurement [36, 37]. WLR has been shown to correlate with age in normotensive subjects [19]. Furthermore retinal areas with higher vessel density have been included in one of our previous studies [37], not allowing direct comparison to the current data.

Skin capillary density previously has been shown to inversely correlate with BP levels in hypertensive patients [9, 10, 38]. This has been predominantly attributed to functional capillary rarefaction [10] due to impaired endothelial function [14]. In cerebral capillaries BP-dependent rarefaction has so far been shown in animal models [16]. Our study group previously described retinal capillary rarefaction in patients with diabetes mellitus type two compared to healthy individuals [21]. Hypertensive patients have been previously shown to present with capillary density similar to diabetic patients [21]. Now we demonstrated for the first time duration of hypertension dependent retinal capillary rarefaction in-vivo in patients with long term and short term hypertension. In our study population systolic and diastolic 24 h ambulatory BP was inversely associated with retinal capillary density indicating a functional BP dependent effect on capillary vessels. But besides the BP-dependent vessel modulation ICD and CapA have been shown to differ between hypertensive and healthy people after adjustment for BP, indicating disease associated changes in functional vessel morphology.

In this analysis we demonstrated an association of ICD and CapA with WLR in our hypertensive study population indicating a correlation of the perfusion dependent functional retinal vessel parameters with the well-established structural vessel parameter WLR. Functional vessel changes are known to frequently precede structural changes. Our hypertensive study population presented with median disease duration of 5 years and no evidence of end-organ damage. We hypothesise that the early stage in hypertension was responsible for not detecting a significant difference in WLR between our hypertensive and healthy study population. Capillary density, a functional and therefore probably earlier parameter was different between both groups and might represent an additional promising early parameter of end-organ damage in hypertensive patients.

Plasma HDL levels show an inverse correlation with incidence of ischemic heart disease and other atherosclerosis-related ischemic conditions [39]. Besides the atheroprotective functions of HDL, attributed to the ability of HDL to uptake cellular cholesterol from peripheral organs and to mediate the transport of excess cholesterol to the liver, HDL has been shown to have various favourable effects on endothelial cells in large vessels, such as coronary arteries [40]. Influence of HDL on capillary density has to our knowledge so far just been investigated in one animal model. In the murine ischemic limb model, HDL promoted angiogenesis and increased the number of histologically detectable capillaries in muscle tissue [39]. We showed in human retinal capillaries an association between HDL and CapA as well as ICD indicating a link of HDL with capillary density. Debbabi and colleagues demonstrated that capillary density in hypertensive patients was influenced by overweight [10]. For this reason adjustment for BMI was made in our study population. After adjustment in partial correlation analysis the association between capillary rarefaction and HDL was even more pronounced.

### Limitations

Our hypertensive study population was selected from a larger group according to quality of SLDF measurements. Nevertheless, since all hypertensive patients showed similar values of age, BMI, gender, duration of disease, blood pressure, heart rate, serum creatinine and serum cholesterol to those of the group with valid evaluation of capillary density (see Additional file 1: Table S1) we are convinced that the selection did not affect the observed associations.

Our control group is of smaller size compared to the hypertensive group, which is a clear limitation of the study. This is a pilot study intended to show first differences in capillary rarefaction between hypertensive and healthy individuals. We plan to increase our control and hypertensive group size in future studies.

Usually 24 h ambulatory BP measurements show lower values compared to office BP values. In our study population systolic 24 h ambulatory BP was similar to systolic office BP in the hypertensive group. A possible reason might be that office BP was taken unsupervised in the present study. Previous studies in treated hypertensive people have shown that systolic office BP measured by automated office BP technique is comparable to, or even lower than daytime ambulatory systolic BP [41].

Antihypertensive treatment is known to have an impact on capillary rarefaction. Skin capillary density was lower in untreated hypertensive subjects compared to normotensive control subjects [1] and antihypertensive treatment has been shown to reverse capillary rarefaction in animals [16, 42] and in humans [10, 43–46] with hypertension. Especially drugs targeting the renin-angiotensin-aldosterone system induce angiogenesis in vivo, probably mediated by activation of bradykinin pathways, resulting in the generation of vascular endothelial growth factor, nitric oxide and, consequently, angiogenesis [47]. In our study population 81% of patients in the hypertensive group received medication targeting the renin-angiotensin-aldosterone system prior to study enrolment. There are data indicating that retinal artery structure already changes after 4 weeks treatment with antihypertensive medication [48]. The included patients of our clinical trials stopped taking antihypertensive medication 4 weeks prior to SLDF measurements. Our results of capillary rarefaction are also in accordance with previous findings obtained in skin capillaries in untreated essential hypertensives [5–7, 49] thereby indicating that there was no bias due to previous antihypertensive treatment.

Our measurements did not allow differentiating between functional and structural capillary rarefaction, since the SLDF measurements used to determine ICD, CapA and RCF are based on perfusion images and reflect the actually perfused capillary bed.

## Conclusions

Retinal capillary rarefaction can be assessed by CapA and ICD in perfusion images using Scanning laser Doppler flowmetry (SLDF). Our measurements of retinal capillary rarefaction and RCF indicate a lower level of retinal capillary density in patients with hypertension stage 1 or 2 compared to normotensive individuals. Additionally, capillary rarefaction was intensified in patients with long time hypertension. Capillary density might represent a promising early parameter of end-organ damage in hypertensive patients. It could be an interesting aim of further investigations to measure capillary rarefaction by using the new technology of adaptive optics [50] imaging.

## Additional file

Additional file 1: Table S1. Patient characteristics of hypertensive study population compared to total hypertensive population. Table including patients characteristics of hypertensive study population compared to total hypertensive population. Hypertensive patients showed no difference in age, BMI, gender, duration of disease, blood pressure, heart rate, serum creatinine and serum cholesterol to those of the group with valid evaluation. (DOCX 28 kb)

## Abbreviations

AU: Arbitrary units
BP: Blood pressure
CA: Cross sectional area
CapA: Capillary area
ICD: Intercapillary distance
ID: Inner vessel diameter
OD: Outer vessel diameter
RCF: Retinal capillary flow
SLDF: Scanning laser Doppler flowmetry
WLR: Wall-to-lumen ratio
WT: Wall thickness

## References

[1] Antonios TF (2006). Microvascular rarefaction in hypertension--reversal or over-correction by treatment?. Am J Hypertens.

[2] Rizzoni D, Porteri E, Boari GE, De Ciuceis C, Sleiman I, Muiesan ML, Castellano M, Miclini M, Agabiti-Rosei E (2003). Prognostic significance of small-artery structure in hypertension. Circulation.

[3] Suzuki K, Masawa N, Sakata N, Takatama M (2003). Pathologic evidence of microvascular rarefaction in the brain of renal hypertensive rats. J Stroke Cerebrovasc Dis.

[4] Paiardi S, Rodella LF, De Ciuceis C, Porteri E, Boari GE, Rezzani R, Rizzardi N, Platto C, Tiberio GA, Giulini SM, Rizzoni D, Agabiti-Rosei E (2009). Immunohistochemical evaluation of microvascular rarefaction in hypertensive humans and in spontaneously hypertensive rats. Clin Hemorheol Microcirc.

[5] Antonios TF, Singer DR, Markandu ND, Mortimer PS, MacGregor GA (1999). Structural skin capillary rarefaction in essential hypertension. Hypertension.

[6] Serne EH, Gans RO, ter Maaten JC, Tangelder GJ, Donker AJ, Stehouwer CD (2001). Impaired skin capillary recruitment in essential hypertension is caused by both functional and structural capillary rarefaction. Hypertension.

[7] Antonios TF, Singer DR, Markandu ND, Mortimer PS, MacGregor GA (1999). Rarefaction of skin capillaries in borderline essential hypertension suggests an early structural abnormality. Hypertension.

[8] Henrich HA, Romen W, Heimgartner W, Hartung E, Baumer F (1988). Capillary rarefaction characteristic of the skeletal muscle of hypertensive patients. Klin Wochenschr.

[9] Cheng C, Diamond JJ, Falkner B (2008). Functional capillary rarefaction in mild blood pressure elevation. Clinical and translational science.

[10] Debbabi H, Uzan L, Mourad JJ, Safar M, Levy BI, Tibirica E (2006). Increased skin capillary density in treated essential hypertensive patients. Am J Hypertens.

[11] Clark MG, Barrett EJ, Wallis MG, Vincent MA, Rattigan S (2002). The microvasculature in insulin resistance and type 2 diabetes. Semin Vasc Med.

[12] Goligorsky MS (2010). Microvascular rarefaction: the decline and fall of blood vessels. Organ.

[13] Schrimpf C, Teebken OE, Wilhelmi M, Duffield JS (2014). The role of pericyte detachment in vascular rarefaction. J Vasc Res.

[14] Cheng C, Daskalakis C, Falkner B (2008). Capillary rarefaction in treated and untreated hypertensive subjects. Ther Adv Cardiovasc Dis.

[15] Ko SH, Cao W, Liu Z (2010). Hypertension management and microvascular insulin resistance in diabetes. Curr Hypertens Rep.

[16] Estato V, Obadia N, Carvalho-Tavares J, Freitas FS, Reis P, Castro-Faria Neto H, Lessa MA, Tibirica E (2013). Blockade of the renin-angiotensin system improves cerebral microcirculatory perfusion in diabetic hypertensive rats. Microvasc Res.

[17] Ritz MF, Fluri F, Engelter ST, Schaeren-Wiemers N, Lyrer PA (2009). Cortical and putamen age-related changes in the microvessel density and astrocyte deficiency in spontaneously hypertensive and stroke-prone spontaneously hypertensive rats. Curr Neurovasc Res.

[18] Rizzoni D, Porteri E, Duse S, De Ciuceis C, Rosei CA, La Boria E, Semeraro F, Costagliola C, Sebastiani A, Danzi P, Tiberio GA, Giulini SM, Docchio F, Sansoni G, Sarkar A, Rosei EA (2012). Relationship between media-to-lumen ratio of subcutaneous small arteries and wall-to-lumen ratio of retinal arterioles evaluated noninvasively by scanning laser doppler flowmetry. J Hypertens.

[19] Harazny JM, Ritt M, Baleanu D, Ott C, Heckmann J, Schlaich MP, Michelson G, Schmieder RE (2007). Increased wall:lumen ratio of retinal arterioles in male patients with a history of a cerebrovascular event. Hypertension.

[20] Rizzoni D, Aalkjaer C, De Ciuceis C, Porteri E, Rossini C, Rosei CA, Sarkar A, Rosei EA (2011). How to assess microvascular structure in humans. High blood pressure & cardiovascular prevention : the official journal of the Italian Society of Hypertension.

[21] Jumar A, Harazny JM, Ott C, Friedrich S, Kistner I, Striepe K, Schmieder RE (2016). Retinal capillary rarefaction in patients with type 2 diabetes mellitus. PLoS One.

[22] Jung F, Pindur G, Ohlmann P, Spitzer G, Sternitzky R, Franke RP, Leithauser B, Wolf S, Park JW (2013). Microcirculation in hypertensive patients. Biorheology.

[23] Jumar A, Ott C, Kistner I, Friedrich S, Michelson G, Harazny JM, Schmieder RE. Early signs of end-organ damage in retinal arterioles in patients with type 2 diabetes compared to hypertensive patients. Microcirculation. 2016; 10.1111/micc.12291 27270643

[24] Michelson G, Welzenbach J, Pal I, Harazny J (1998). Automatic full field analysis of perfusion images gained by scanning laser doppler flowmetry. Br J Ophthalmol.

[25] Weber T, Wassertheurer S, Rammer M, Haiden A, Hametner B, Eber B (2012). Wave reflections, assessed with a novel method for pulse wave separation, are associated with end-organ damage and clinical outcomes. Hypertension.

[26] Weber T, Wassertheurer S, Rammer M, Maurer E, Hametner B, Mayer CC, Kropf J, Eber B (2011). Validation of a brachial cuff-based method for estimating central systolic blood pressure. Hypertension.

[27] Harazny JM, Raff U, Welzenbach J, Ott C, Ritt M, Lehmann M, Michelson G, Schmieder RE (2011). New software analyses increase the reliability of measurements of retinal arterioles morphology by scanning laser doppler flowmetry in humans. J Hypertens.

[28] Jumar A, Harazny JM, Ott C, Kistner I, Friedrich S, Schmieder RE. Improvement in retinal capillary rarefaction after valsartan treatment in hypertensive patients. J Clin Hypertens. 2016; 10.1111/jch.12851 PMC8031650 27306560

[29] Ritt M, Schmieder RE (2009). Wall-to-lumen ratio of retinal arterioles as a tool to assess vascular changes. Hypertension.

[30] Kreis AJ, Nguyen T, Rogers S, Wang JJ, Harazny J, Michelson G, Farouque HM, Wong TY (2008). Reliability of different image analysis methods for scanning laser doppler flowmetry. Curr Eye Res.

[31] Michelson G, Schmauss B, Langhans MJ, Harazny J, Groh MJ (1996). Principle, validity, and reliability of scanning laser doppler flowmetry. J Glaucoma.

[32] Michelson G, Welzenbach J, Pal I, Harazny J (2001). Functional imaging of the retinal microvasculature by scanning laser doppler flowmetry. Int Ophthalmol.

[33] Muiesan ML, Salvetti M, Rizzoni D, Paini A, Agabiti-Rosei C, Aggiusti C, Agabiti Rosei E (2013). Resistant hypertension and target organ damage. Hypertension research : official journal of the Japanese Society of Hypertension.

[34] Ritt M, Harazny JM, Ott C, Schneider MP, Schlaich MP, Michelson G, Schmieder RE (2009). Wall-to-lumen ratio of retinal arterioles is related with urinary albumin excretion and altered vascular reactivity to infusion of the nitric oxide synthase inhibitor n-monomethyl-l-arginine. J Hypertens.

[35] Ritt M, Harazny JM, Ott C, Raff U, Lehmann M, Michelson G, Schmieder RE (2012). Influence of blood flow on arteriolar wall-to-lumen ratio in the human retinal circulation in vivo. Microvasc Res.

[36] Ritt M, Harazny JM, Ott C, Schlaich MP, Schneider MP, Michelson G, Schmieder RE (2008). Analysis of retinal arteriolar structure in never-treated patients with essential hypertension. J Hypertens.

[37] Delles C, Michelson G, Harazny J, Oehmer S, Hilgers KF, Schmieder RE (2004). Impaired endothelial function of the retinal vasculature in hypertensive patients. Stroke.

[38] Triantafyllou A, Anyfanti P, Pyrpasopoulou A, Triantafyllou G, Aslanidis S, Douma S (2015). Capillary rarefaction as an index for the microvascular assessment of hypertensive patients. Curr Hypertens Rep.

[39] Sumi M, Sata M, Miura S, Rye KA, Toya N, Kanaoka Y, Yanaga K, Ohki T, Saku K, Nagai R (2007). Reconstituted high-density lipoprotein stimulates differentiation of endothelial progenitor cells and enhances ischemia-induced angiogenesis. Arterioscler Thromb Vasc Biol.

[40] Barter PJ, Nicholls S, Rye KA, Anantharamaiah GM, Navab M, Fogelman AM (2004). Antiinflammatory properties of hdl. Circ Res.

[41] Kjeldsen SE, Lund-Johansen P, Nilsson PM, Mancia G. Unattended blood pressure measurements in the systolic blood pressure intervention trial: implications for entry and achieved blood pressure values compared with other trials. Hypertension. 2016; 10.1161/HYPERTENSIONAHA.116.07257 27001295

[42] Sabino B, Lessa MA, Nascimento AR, Rodrigues CA, Henriques M, Garzoni LR, Levy BI, Tibirica E (2008). Effects of antihypertensive drugs on capillary rarefaction in spontaneously hypertensive rats: Intravital microscopy and histologic analysis. J Cardiovasc Pharmacol.

[43] Tsioufis C, Dimitriadis K, Katsiki N, Tousoulis D (2015). Microcirculation in hypertension: an update on clinical significance and therapy. Curr Vasc Pharmacol.

[44] Aellen J, Dabiri A, Heim A, Liaudet L, Burnier M, Ruiz J, Feihl F, Waeber B (2012). Preserved capillary density of dorsal finger skin in treated hypertensive patients with or without type 2 diabetes. Microcirculation.

[45] Kaiser SE, Sanjuliani AF, Estato V, Gomes MB, Tibirica E (2013). Antihypertensive treatment improves microvascular rarefaction and reactivity in low-risk hypertensive individuals. Microcirculation.

[46] Agabiti-Rosei E. [structural and functional changes of the microcirculation in hypertension: Influence of pharmacological therapy]. *Drugs*. 2003;63 Spec No 1:19–29. 12708883

[47] Battegay EJ, de Miguel LS, Petrimpol M, Humar R (2007). Effects of anti-hypertensive drugs on vessel rarefaction. Curr Opin Pharmacol.

[48] De Ciuceis C, Salvetti M, Rossini C, Muiesan ML, Paini A, Duse S, La Boria E, Semeraro F, Cancarini A, Rosei CA, Sarkar A, Ruggeri G, Caimi L, Ricotta D, Rizzoni D, Rosei EA (2014). Effect of antihypertensive treatment on microvascular structure, central blood pressure and oxidative stress in patients with mild essential hypertension. J Hypertens.

[49] Antonios TF, Rattray FE, Singer DR, Markandu ND, Mortimer PS, MacGregor GA (1999). Maximization of skin capillaries during intravital video-microscopy in essential hypertension: comparison between venous congestion, reactive hyperaemia and core heat load tests. Clin Sci (Lond).

[50] Koch E, Rosenbaum D, Brolly A, Sahel JA, Chaumet-Riffaud P, Girerd X, Rossant F, Paques M (2014). Morphometric analysis of small arteries in the human retina using adaptive optics imaging: relationship with blood pressure and focal vascular changes. J Hypertens.

