# Supplementary material for: Retinal capillary rarefaction in patients with untreated mild-moderate hypertension
Source: BMC Cardiovasc Disord. 2017 Dec 21;17:300. doi: 10.1186/s12872-017-0732-x (PMC5740840; doi:10.1186/s12872-017-0732-x)
Supplement: Additional file 1: Table S1. — Patient characteristics of hypertensive study population compared to total hypertensive population. Table including patients characteristics of hypertensive study population compared to total hypertensive population. Hypertensive patients showed no difference in age, BMI, gender, duration of disease, blood pressure, heart rate, serum creatinine and serum cholesterol to those of the group with valid evaluation. (DOCX 28 kb) [file 12872_2017_732_MOESM1_ESM.docx]

**Supplement Table 1: Patient characteristics of hypertensive study population compared to total hypertensive population**

|  | Study population | Total population |
| --- | --- | --- |
| Age (years) | 52 ± 11 | 52 ± 11 |
| BMI (kg/m^2^) | 28.1 ± 3.6 | 28.0 ± 3.4 |
| Sex (male/female) | 99/35 | 123/35 |
| Duration disease (month) | 67  (24 – 133) | 68  (31-140) |
| Systolic Office BP (mmHg) | 146 ± 12 | 148 ± 13 |
| Diastolic Office BP (mmHg) | 90 ± 8.8 | 92 ± 8.8 |
| HR (bpm) | 72 ± 10.3 | 72 ± 10 |
| Systolic 24h Ambulatory BP (mmHg) | 145 ± 10.4 | 146 ± 9.6 |
| Diastolic 24h Ambulatroy BP (mmHg) | 90 ± 9.7 | 92 ± 9.6 |
| Serum creatinine (mmol/L) | 0.079 ± 0.01 | 0.073 ± 0.01 |
| LDL (mmol/L) | 3.81 ± 0.91 | 3.74 ± 0.75 |
| HDL (mmol/L) | 1.35 ± 0.31 | 1.27 ± 0.29 |

Data are given as mean ± SD, duration of disease is given as median and interquartile range, BMI – body mass index, BP – blood pressure, HR – heart rate, LDL – low density lipids, HDL – high density lipids
